# Supplementary material for: A metatranscriptomic exploration of fungal and bacterial contributions to allochthonous leaf litter decomposition in the streambed
Source: PeerJ. 2025 Apr 25;13:e19120. doi: 10.7717/peerj.19120 (PMC12036579; doi:10.7717/peerj.19120)
Supplement: Supplemental Information 1 [file peerj-13-19120-s001.docx]

**Supplementary Information for**

**A metatranscriptomic exploration of fungal and bacterial contributions to allochthonous leaf litter decomposition in the streambed**

Aman Deep^1,6^, Guido Sieber^1,3^, Lisa Boden^1^, Gwendoline M. David^5^, Daria Baikova^4^, Dominik Buchner^7^, Jörn Starke^2^, Tom L. Stach^2,3^, Torben Reinders^1^, Una Hadžiomerović^3,4^, Sára Beszteri^1^, Alexander J. Probst^2,3,8,^ , Jens Boenigk^1,3^, Daniela Beisser^6,3^

1. Biodiversity, Faculty of Biology, University of Duisburg-Essen, Essen, Germany

2. Environmental Metagenomics, Research Center One Health Ruhr of the University Alliance Ruhr,

Faculty of Chemistry, University of Duisburg-Essen, Essen, Germany

3. Centre of Water and Environmental Research (ZWU), University of Duisburg-Essen, Essen,

Germany

4. Aquatic Microbiology, Environmental Microbiology and Biotechnology, Faculty of Chemistry,

University of Duisburg-Essen, Essen, Germany

5. Leibniz Institute of Freshwater Ecology and Inland Fisheries, Department of Plankton and

Microbial Ecology, Stechlin, Germany

6. Department of Engineering and Natural Sciences, Westphalian University of Applied Sciences,

Recklinghausen, Germany

7. Aquatic Ecosystem Research, University of Duisburg-Essen, Essen, Germany

8. Centre of Medical Biotechnology (ZMB), University of Duisburg-Essen, Essen, Germany

# Methods:

Leaf discs analysis:

For the leaf discs, two bags (*i.e.,* 6 leaf discs) were collected in each flume, stored in boxes with flume water, and processed directly at arrival in the laboratory. Leaf discs were cleaned and placed in petri dishes filled with 15 mL of filtered flume water. The incubation took place on an orbital shaker (50 rpm) at 12 ± 1 ◦C and a photoperiod of 4 h light, 12 h dark and 8 h light for a total of 24 h. The resulting spore suspension was decanted, the leaf discs and Petri dish rinsed with 4 mL of filtered flume water, and the suspension was fixed with 1 ml of 37 % formalin. Later, the suspension was gently mixed to ensure a homogeneous spore distribution before filtering a 5 mL or 7.5 mL aliquot on a 5 μm pore size cellulose nitrate membrane filter (Whatman) and staining the spores with 0.1 % trypan blue in 60 % lactic acid (Gessner *et al*., 2003). Between 12 to 100 % of the filter surface depending on spore density was screened (200x and 400x magnification) to count and identify the spores using primarily the key by Gulis *et al*., (2019). Sporulation rates are expressed as the number of spores released per leaf disc per day. The fungal taxa from amplicon were compared to the ITS rDNA barcodes database of aquatic hyphomycetes (Franco-Duarte *et al*., 2022).

# Results:

Active fungal species from sporulation assay:

Sporulation activity was detected on the leaf litter discs, with a total of 10 fungal species observed (Figure S8). Two species dominated the sporulating communities on the leaves in the three control flumes, an unidentified species (sigmoid spores 60-70 µm length) and *Tetracladium marchalianum*.

# Figures:
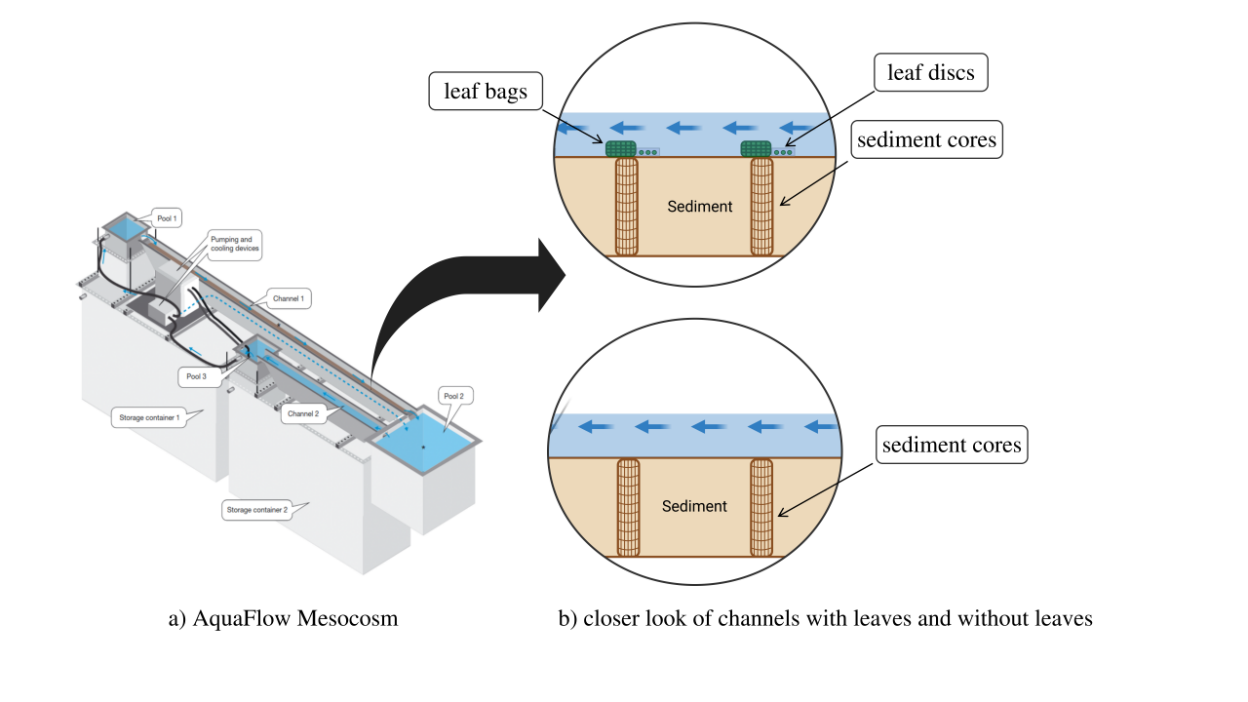


**Figure S1:** Schematic diagram of AquaFlow mesocosm experimental setup.


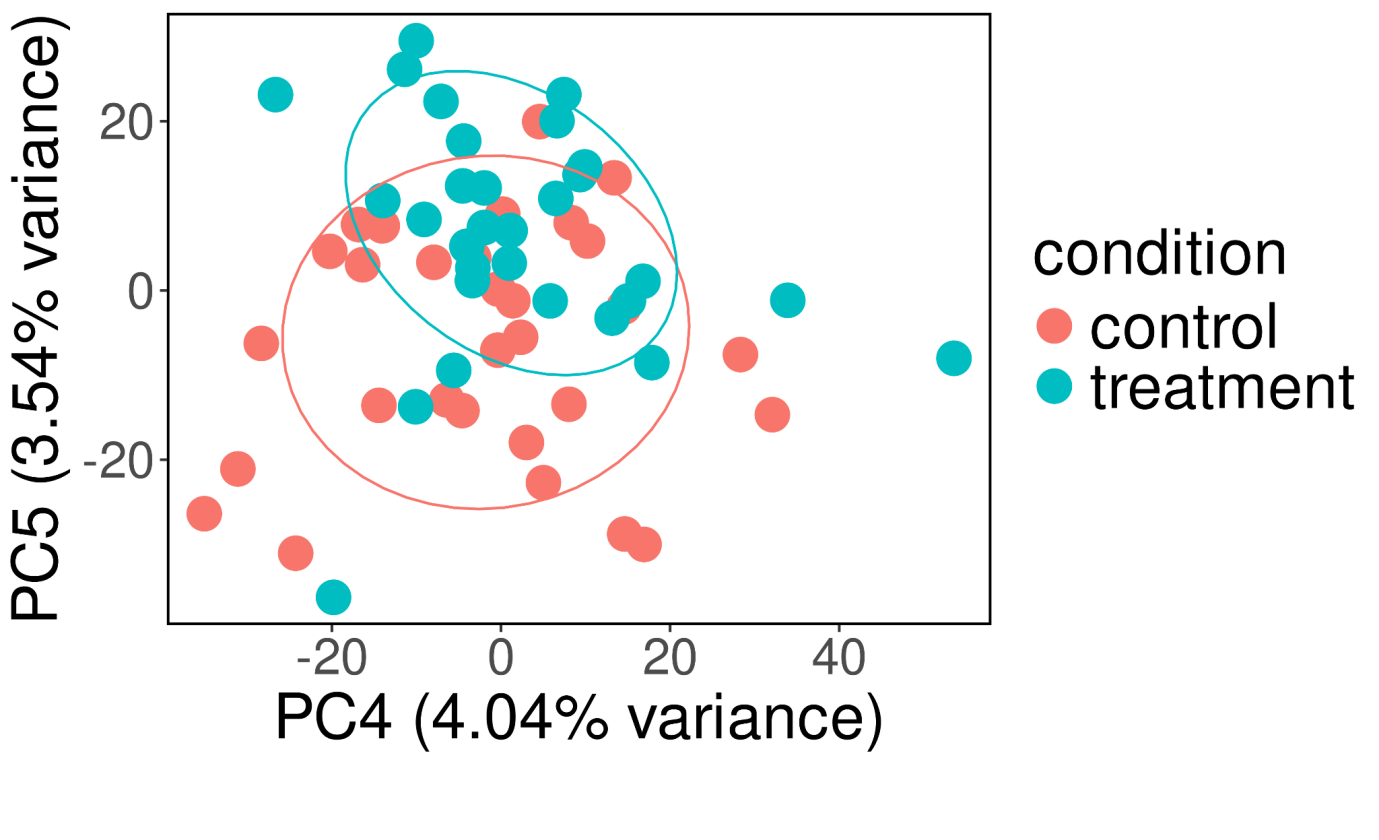


**Figure S2:** Principal component analysis of bacterial KEGG Orthologs (KOs) in sediment samples. Enzyme expressions were transformed with varianceStabilizingTransformation from DESeq2 and principal components were calculated with prcomp. Principal components 4 and 5 are shown as X and Y axes with their explained variances. Samples are coloured by condition: control (red) and treatment (cyan) and ellipses highlight the control and treatment groups (ellipse level=0.75). Only at higher axes with a small explained variance, a separation between control and treatment is visible.


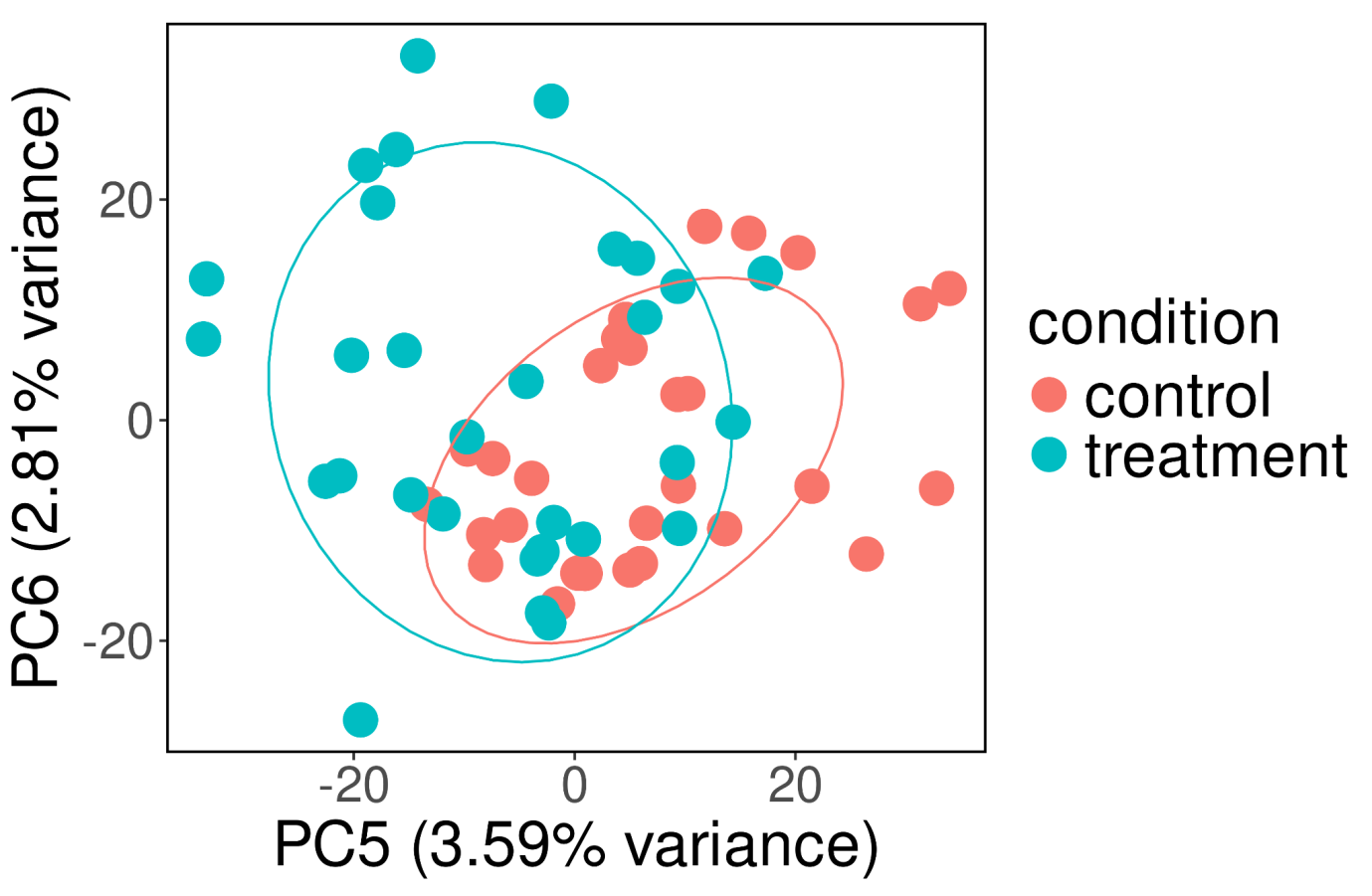


**Figure S3**: Principal component analysis of fungal KEGG Orthologs (KOs) in sediment samples. Enzyme expressions were transformed with varianceStabilizingTransformation from DESeq2 and principal components were calculated with prcomp. Principal components 5 and 6 are shown as X and Y axes with their explained variances. Samples are coloured by condition: control (red) and treatment (cyan) and ellipses highlight the control and treatment groups (ellipse level=0.75). Only at higher axes with a small explained variance, a separation between control and treatment is visible.

**
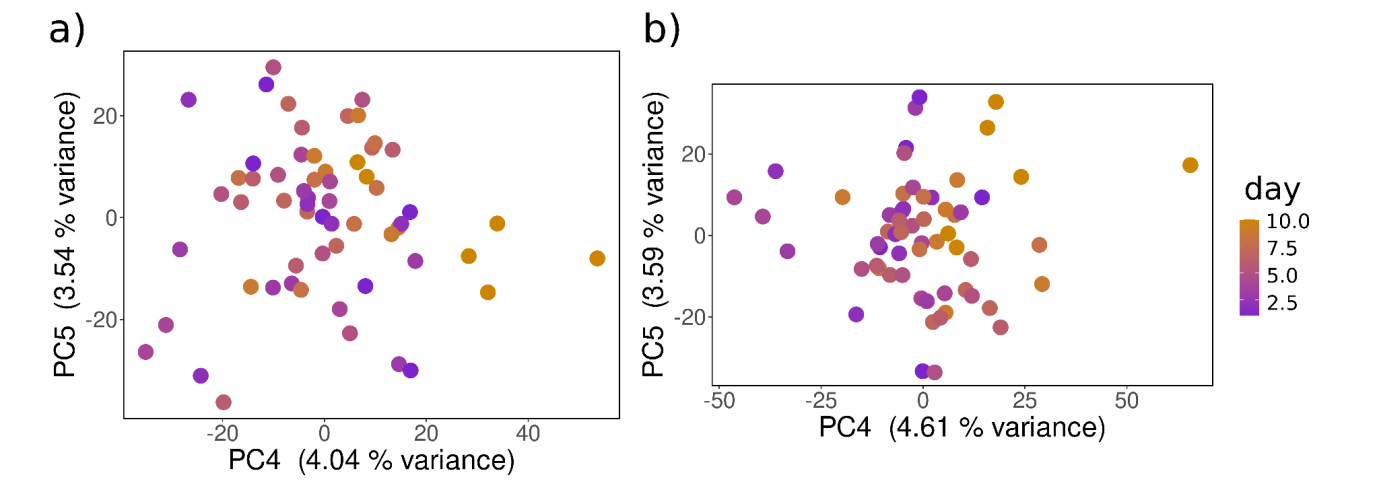
**

**Figure S4**: Principal component analysis of a) bacterial and b) fungal KEGG Orthologs (KOs) in sediment samples. Enzyme expressions were transformed with varianceStabilizingTransformation from DESeq2 and principal components were calculated with prcomp. Principal components 4 and 5 are shown as X and Y axes with their explained variances. Samples are coloured with gradient by day: day1 (purple) and day10 (orange). Only at higher axes with a small explained variance, a separation between timepoints is visible.


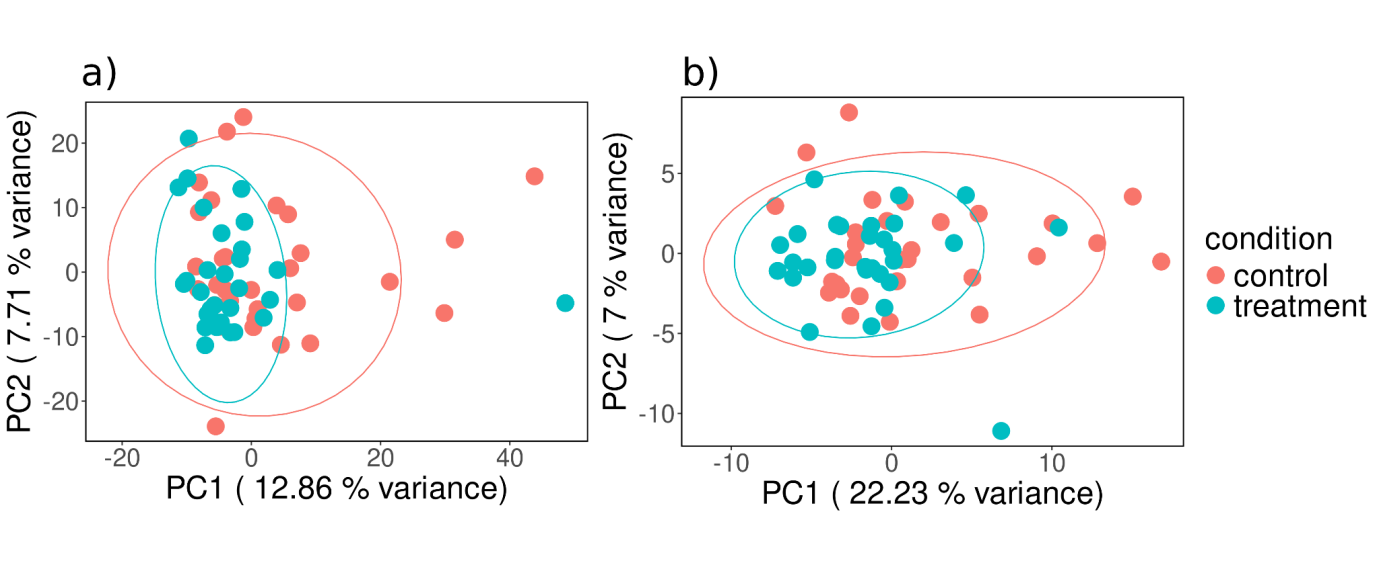


**Figure S5:** Principal Component Analysis of a) bacterial and b) fungal OTUs in sediment samples. OTUs abundance were transformed with varianceStabilizingTransformation from DESeq2 and principal components were calculated with prcomp. Principal components 6 and 7 are shown as X and Y axes with their explained variances for bacteria. Principal components 5 and 6 are shown as X and Y axes with their explained variances for fungi. Samples are coloured by condition: control (red) and treatment (cyan) and ellipses highlight the control and treatment groups (ellipse level =0.75).


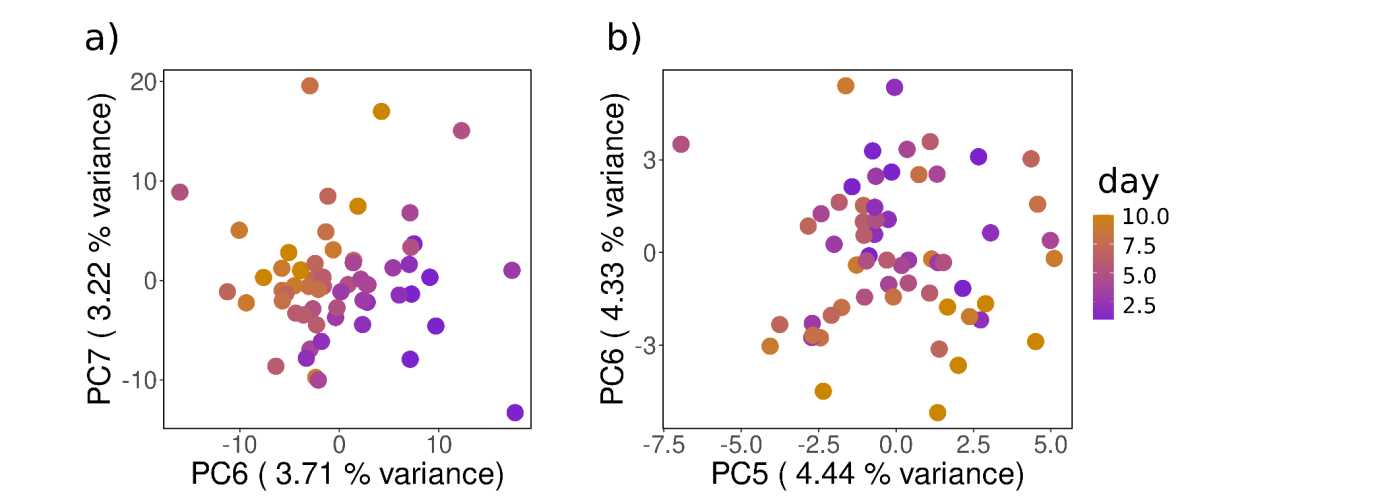


**Figure S6**: Principal component analysis of a) bacterial and b) fungal OTUs in sediment samples. OTUs abundance were transformed with varianceStabilizingTransformation from DESeq2 and principal components were calculated with prcomp. Principal components 6 and 7 are shown as X and Y axes with their explained variances for bacteria. Principal components 5 and 6 are shown as X and Y axes with their explained variances for fungi. Samples are coloured with a gradient by day: day 1 purple and day 10 orange. The time effect is visible only at higher axes with a small explained variance.


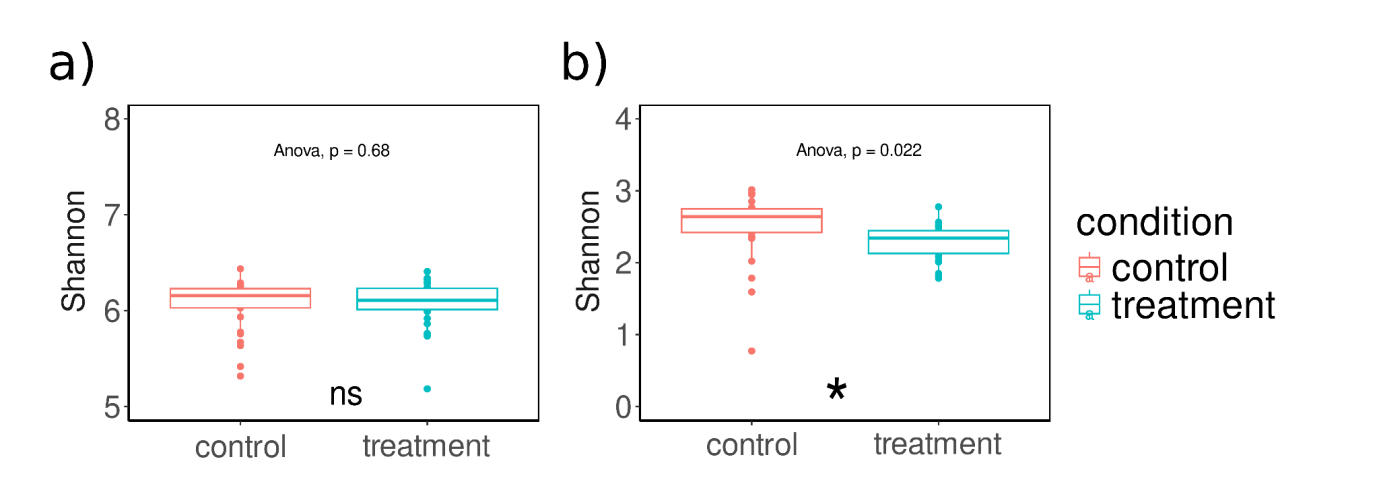


**Figure S7**: Alpha diversity analysis for a) bacteria and b) fungi with control vs treatment. Shannon indices were calculated between control and treatment and ANOVA was performed for significance. Anova with a p-value minimum and lower than 0.05 implied as significant (asterisk:*) and higher as non-significant (ns).


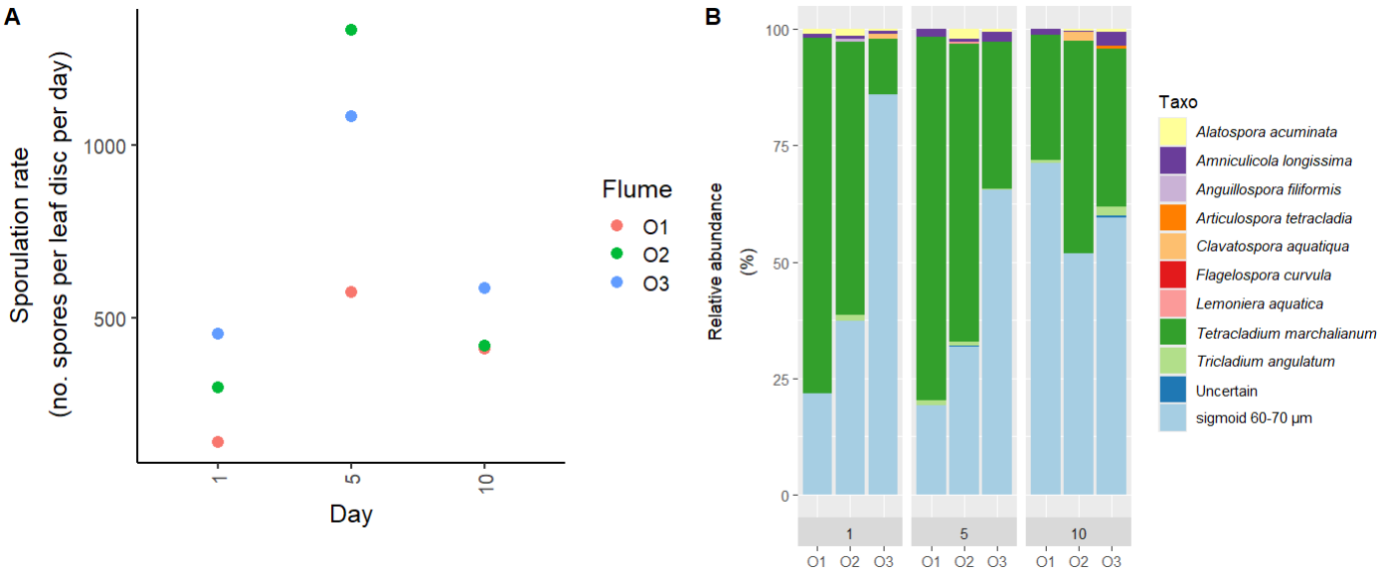


**Figure S8:** Sporulation rates (A) and relative abundance (B) of the aquatic hyphomycetes communities identified from the leaf-litter bags of the treatment flumes. The sampling days were D1, D5 and D10. The label O1, O2, and O3 correspond to the three replicated flumes.

# Tables:

| PCR phase | Time | 1st PCR-Temperature [°C] 20 cycles | 2nd PCR-Temperature [°C] 25 cycles |
| --- | --- | --- | --- |
| Initial denaturation | 5 min | 95 | 95 |
| Denaturation (25 cycles) | 30 s | 95 | 95 |
| Annealing (25 cycles) | 90 s | 50 | 61 |
| Elongation (25 cycles) | 30 s | 72 | 72 |
| Final elongation | 10 min | 68 | 68 |

**Table S1**: Cycling condition for two-step PCR approach for 16S and ITS gene amplification.

**supplementary_table_S2.xlsx**

**Table S2**: Summary of Sequencing Data – Number of Reads, Assembled Contigs, and Reads Annotated to Bacteria and Fungi

| **Bacteria** |  |  |  |
| --- | --- | --- | --- |
|  | R2 | F | Pr(>F) |
| condition | 0.02 | 1.48 | 0.17 |
| day | 0.02 | 1.58 | 0.15 |
| condition:day | 0.01 | 0.63 | 0.63 |
| **Fungi** |  |  |  |
|  | R2 | F | Pr(>F) |
| condition | 0.02 | 0.98 | 0.44 |
| day | 0.02 | 1.38 | 0.23 |
| condition:day | 0.02 | 1.35 | 0.27 |

**Table S3:** A non-parametric statistical permutational test (PERMANOVA) for bacterial and fungal KOs. Adonis2 was performed separately on the Bray Curtis distance matrix of all KOs belonging to bacteria and fungi.

| **KO ID** | **CAZyme family** | **EC numbers and descriptions** |
| --- | --- | --- |
| K00702 | GT36 | 2.4.1.20: cellobiose phosphorylase |
| K01178 | GH15 | 3.2.1.3: glucan 14-alpha-glucosidase |
| K01179 | GH5,GH9 | 3.2.1.4: cellulase |
| K01186 | GH33 | 3.2.1.18: exo-alpha-sialidase |
| K01188 | GH1 | 3.2.1.21: beta-glucosidase |
| K01190 | GH2 | 3.2.1.23: beta-galactosidase |
| K01198 | GH43 | 3.2.1.37: xylan 14-beta-xylosidase |
| K01209 | GH51 | 3.2.1.55: non-reducing end alpha-L-arabinofuranosidase |
| K01213 | GH28 | 3.2.1.67: galacturonan 14-alpha-galacturonidase |
| K01214 | GH13,CBM48 | 3.2.1.68: isoamylase |
| K01216 | GH16 | 3.2.1.73: licheninase |
| K01218 | GH26 | 3.2.1.78: mannan endo-14-beta-mannosidase |
| K01225 | GH7 | 3.2.1.91: cellulose 14-beta-cellobiosidase (non-reducing end) |
| K05349 | GH3 | 3.2.1.21: beta-glucosidase |
| K06113 | GH43 | 3.2.1.99: arabinan endo-15-alpha-L-arabinosidase |
| K07406 | GH4 | 3.2.1.22: alpha-galactosidase |
| K12111 | GH2 | 3.2.1.23: beta-galactosidase |
| K12308 | GH42 | 3.2.1.23: beta-galactosidase |
| K12309 | GH35 | 3.2.1.23: beta-galactosidase |
| K12357 | GH33 | 3.2.1.18: exo-alpha-sialidase |
| K15920 | GH3 | 3.2.1.37: xylan 14-beta-xylosidase |
| K15921 | CBM6,GH43 | 3.2.1.55: non-reducing end alpha-L-arabinofuranosidase |
| K18195 | PL4 | 4.2.2.23: rhamnogalacturonan endolyase |
| K18197 | PL11 | 4.2.2.23: rhamnogalacturonan endolyase |
| K18198 | PL11 | 4.2.2.24: rhamnogalacturonan exolyase |
| K18579 | GH5 | 3.2.1.164: galactan endo-16-beta-galactosidase |
| K18580 | GH28 | 3.2.1.171: rhamnogalacturonan hydrolase |
| K18650 | GH28 | 3.2.1.82: exo-poly-alpha-digalacturonosidase |
| K19247 | GH83 | 3.2.1.18: exo-alpha-sialidase |
| K19357 | GH7 | 3.2.1.4: cellulase |
| K19392 | GH34 | 3.2.1.18: exo-alpha-sialidase |
| K19668 | GH6 | 3.2.1.91: cellulose 14-beta-cellobiosidase (non-reducing end) |
| K20542 | GH8 | 3.2.1.4: cellulase |
| K20832 | GH16 | 3.2.1.181: galactan endo-beta-13-galactanase |
| K20844 | CBM42,GH54 | 3.2.1.55: non-reducing end alpha-L-arabinofuranosidase |
| K22268 | GH52 | 3.2.1.37: xylan 14-beta-xylosidase |
| K00037 |  | 1.1.1.50: 3alpha-hydroxysteroid 3-dehydrogenase (Si-specific).,1.1.1.357: 3alpha-hydroxysteroid 3-dehydrogenase.,1.1.1.225: chlordecone reductase |
| K00089 |  | 1.1.1.213: 3alpha-hydroxysteroid dehydrogenase (Re-specific).,1.1.1.357: 3alpha-hydroxysteroid 3-dehydrogenase |
| K00212 |  | 1.1.1.149: 20alpha-hydroxysteroid dehydrogenase.,1.1.1.357: 3alpha-hydroxysteroid 3-dehydrogenase.,1.3.1.20: trans-12-dihydrobenzene-12-diol dehydrogenase |
| K00344 |  | 1.6.5.5: NAPH:quinone reductase |
| K00359 |  | 1.6.5.5: NAPH:quinone reductase |
| K00421 |  | 1.10.3.2: laccase |
| K00446 |  | 1.13.11.2: catechol 23-dioxygenase |
| K01046 |  | 3.1.1.3: triacylglycerol lipase |
| K01051 |  | 3.1.1.11: pectinesterase |
| K01181 |  | 3.2.1.8: endo-14-beta-xylanase |
| K01184 |  | 3.2.1.15: endo-polygalacturonase |
| K01189 |  | 3.2.1.22: alpha-galactosidase |
| K01224 |  | 3.2.1.89: arabinogalactan endo-beta-14-galactanase |
| K01235 |  | 3.2.1.139: alpha-glucuronidase |
| K01728 |  | 4.2.2.2: pectate lyase |
| K01731 |  | 4.2.2.9: pectate disaccharide-lyase |
| K01732 |  | 4.2.2.10: pectin lyase |
| K03823 |  | 2.3.1.183: phosphinothricin acetyltransferase |
| K04119 |  | 1.1.1.51: 3(or 17)beta-hydroxysteroid dehydrogenase.,1.1.1.188: prostaglandin-F synthase.,1.1.1.213: 3alpha-hydroxysteroid dehydrogenase (Re-specific).,1.1.1.357: 3alpha-hydroxysteroid 3-dehydrogenase |
| K04462 |  | 2.1.1.367: [histone H3]-lysine(9) N-methyltransferase |
| K05350 |  | 3.2.1.21: beta-glucosidase |
| K05884 |  | 1.1.1.337: L-2-hydroxycarboxylate dehydrogenase (NA(+)) |
| K05895 |  | 1.3.1.54: precorrin-6A reductase.,1.3.1.106: cobalt-precorrin-6A reductase |
| K05909 |  | 1.10.3.2: laccase |
| K05972 |  | 3.1.1.72: acetylxylan esterase |
| K06957 |  | 2.3.1.193: tRNA(Met) cytidine acetyltransferase |
| K07104 |  | 1.13.11.2: catechol 23-dioxygenase |
| K07407 |  | 3.2.1.22: alpha-galactosidase |
| K08764 |  | 2.3.1.176: propanoyl-CoA C-acyltransferase |
| K11424 |  | 2.1.1.357: [histone H3]-lysine(36) N-dimethyltransferase |
| K11426 |  | 2.1.1.354: [histone H3]-lysine(4) N-trimethyltransferase.,2.1.1.357: [histone H3]-lysine(36) N-dimethyltransferase |
| K11433 |  | 2.1.1.357: [histone H3]-lysine(36) N-dimethyltransferase |
| K12298 |  | 3.1.1.3: triacylglycerol lipase.,3.1.1.13: sterol esterase |
| K12925 |  | 2.3.1.166: 2alpha-hydroxytaxane 2-O-benzoyltransferase |
| K12936 |  | 2.3.1.153: anthocyanin 5-(6'''-hydroxycinnamoyltransferase) |
| K13065 |  | 2.3.1.133: shikimate O-hydroxycinnamoyltransferase |
| K13231 |  | 2.3.1.146: pinosylvin synthase |
| K13465 |  | 3.2.1.8: endo-14-beta-xylanase |
| K13534 |  | 3.1.1.3: triacylglycerol lipase |
| K13616 |  | 3.1.1.3: triacylglycerol lipase |
| K14073 |  | 3.1.1.3: triacylglycerol lipase |
| K14074 |  | 3.1.1.3: triacylglycerol lipase |
| K14075 |  | 3.1.1.3: triacylglycerol lipase |
| K14076 |  | 3.1.1.3: triacylglycerol lipase |
| K14080 |  | 2.1.1.246: [methyl-Co() methanol-specific corrinoid protein]:coenzyme M,2.1.1.377: [methyl-Co() glycine betaine-specific corrinoid protein]--coenzyme M |
| K14452 |  | 3.1.1.3: triacylglycerol lipase |
| K14674 |  | 3.1.1.3: triacylglycerol lipase.,3.1.1.13: sterol esterase.,3.1.1.4: phospholipase A2.,2.3.1.51: 1-acylglycerol-3-phosphate O-acyltransferase |
| K14675 |  | 3.1.1.3: triacylglycerol lipase |
| K14759 |  | 5.4.4.2: isochorismate synthase.,2.2.1.9: 2-succinyl-5-enolpyruvyl-6-hydroxy-3-cyclohexene-1-carboxylic-acid,4.2.99.20: 2-succinyl-6-hydroxy-24-cyclohexadiene-1-carboxylate synthase.,4.2.1.113: o-succinylbenzoate synthase |
| K15530 |  | 3.1.1.86: rhamnogalacturonan acetylesterase |
| K15588 |  | 2.1.1.357: [histone H3]-lysine(36) N-dimethyltransferase |
| K15873 |  | 1.3.1.116: 7beta-hydroxy-3-oxochol-24-oyl-CoA 4-desaturase |
| K16213 |  | 5.1.3.11: cellobiose epimerase |
| K16816 |  | 3.1.1.3: triacylglycerol lipase |
| K17743 |  | 1.1.1.307: -xylose reductase [NA(P)H] |
| K17900 |  | 3.1.1.3: triacylglycerol lipase |
| K18337 |  | 1.1.1.378: L-rhamnose 1-dehydrogenase [NA(P)(+)].,1.1.1.377: L-rhamnose 1-dehydrogenase (NAP(+)).,1.1.1.173: L-rhamnose 1-dehydrogenase |
| K19068 |  | 1.1.1.367: UP-2-acetamido-26-beta-L-arabino-hexul-4-ose reductase |
| K19069 |  | 1.1.99.18: cellobiose dehydrogenase (acceptor) |
| K19355 |  | 3.2.1.78: mannan endo-14-beta-mannosidase |
| K19551 |  | 4.2.2.2: pectate lyase.,4.2.2.10: pectin lyase |
| K19569 |  | 2.1.1.307: 8-demethyl-8-(23-dimethoxy-alpha-L-rhamnosyl)-tetracenomycin-C 4'-O- |
| K19639 |  | 1.1.1.317: perakine reductase |
| K19649 |  | 1.1.1.327: 5-exo-hydroxycamphor dehydrogenase |
| K19653 |  | 1.1.1.347: geraniol dehydrogenase (NA(+)) |
| K19711 |  | 1.1.1.387: L-serine 3-dehydrogenase (NA(+)) |
| K19811 |  | 1.1.1.357: 3alpha-hydroxysteroid 3-dehydrogenase |
| K19861 |  | 2.3.1.196: benzyl alcohol O-benzoyltransferase.,2.3.1.232: methanol O-anthraniloyltransferase |
| K20205 |  | 1.11.1.13: manganese peroxidase |
| K20238 |  | 2.1.1.317: sphingolipid C(9)-methyltransferase |
| K20939 |  | 1.1.1.397: beta-methylindole-3-pyruvate reductase |
| K21103 |  | 2.1.1.327: phenazine-1-carboxylate N-methyltransferase |
| K21580 |  | 2.1.1.337: reticuline N-methyltransferase |
| K21839 |  | 1.3.1.103: 2-haloacrylate reductase |
| K22247 |  | 2.3.1.136: polysialic-acid O-acetyltransferase |
| K22283 |  | 3.1.1.3: triacylglycerol lipase |
| K22284 |  | 3.1.1.3: triacylglycerol lipase |
| K22410 |  | 2.1.1.367: [histone H3]-lysine(9) N-methyltransferase |
| K22462 |  | 1.3.1.113: (4-alkanoyl-5-oxo-25-dihydrofuran-3-yl)methyl phosphate reductase |
| K22539 |  | 4.2.2.2: pectate lyase |
| K22577 |  | 2.1.1.347: (+)-O-methylkolavelool synthase |
| K22933 |  | 3.2.1.67: galacturonan 14-alpha-galacturonidase |
| K22994 |  | 4.2.2.9: pectate disaccharide-lyase |
| K23515 |  | 1.11.1.14: lignin peroxidase |
| K23530 |  | 4.2.3.201: hydropyrene synthase.,4.2.3.202: hydropyrenol synthase.,4.2.3.203: isoelisabethatriene synthase |
| K23550 |  | 3.2.1.18: exo-alpha-sialidase |
| K23968 |  | 1.3.1.123: 7-epi-iridoid synthase |
| K25108 |  | 3.2.1.73: licheninase |
| K20547 | CBM18,GH19 | 3.2.1.14: chitinase |
| K01176 | cazy:GH13 | 3.2.1.1: alpha-amylase |
| K01177 | cazy:GH14 | 3.2.1.2: beta-amylase |
| K01183 | cazy:GH18 | 3.2.1.14: chitinase |
| K01200 | cazy:GH13,CBM48 | 3.2.1.41: pullulanase |
| K01207 |  | 3.2.1.52: beta-N-acetylhexosaminidase |
| K05343 | cazy:GH13 | 3.2.1.1: alpha-amylase |
| K07405 | cazy:GH57 | 3.2.1.1: alpha-amylase |
| K10759 |  | 3.1.1.20: tannase |
| K12047 | cazy:GH31 | 3.2.1.3: glucan 14-alpha-glucosidase |
| K12373 | cazy:GH20 | 3.2.1.52: beta-N-acetylhexosaminidase |
| K13381 | cazy:CBM15,GH18 | 3.2.1.14: chitinase |
| K14459 | cazy:GH20 | 3.2.1.52: beta-N-acetylhexosaminidase |
| K20730 | cazy:GH20 | 3.2.1.52: beta-N-acetylhexosaminidase |
| K21574 | cazy:GH97 | 3.2.1.3: glucan 14-alpha-glucosidase |

**Table S4:** List of KEGG Orthologs, CAZyme families, EC numbers and enzyme names involved in CPOM degradation.

| **KEGG_ID** | Sum_Sq | Mean_Sq | F_value | Pr_F |
| --- | --- | --- | --- | --- |
| K00421 | 128961.33 | 128961.33 | 9.67 | 0.01 |
| K01179 | 734472.00 | 734472.00 | 120.42 | 0.00 |
| K01181 | 327360.33 | 327360.33 | 11.04 | 0.01 |
| K01183 | 7500.00 | 7500.00 | 5.56 | 0.04 |
| K01188 | 1496.33 | 1496.33 | 5.56 | 0.04 |
| K01200 | 4704.50 | 4704.50 | 7.63 | 0.01 |
| K01207 | 81675.00 | 81675.00 | 6.64 | 0.03 |
| K01732 | 27840.33 | 27840.33 | 10.74 | 0.01 |
| K05349 | 255792.00 | 255792.00 | 7.33 | 0.02 |
| K05350 | 5547.00 | 5547.00 | 6.17 | 0.03 |
| K05909 | 752001.33 | 752001.33 | 6.53 | 0.03 |
| K12309 | 24.00 | 24.00 | 13.09 | 0.02 |
| K18337 | 1176.00 | 1176.00 | 23.13 | 0.01 |
| K19551 | 4408.33 | 4408.33 | 43.97 | 0.00 |
| K19668 | 18252.00 | 18252.00 | 17.41 | 0.00 |
| K21574 | 1496.33 | 1496.33 | 6.05 | 0.03 |
|  |  |  |  |  |
| Day 6 |  |  |  |  |
| K01179 | 305762 | 305762 | 5.429928699 | 0.033208612 |
| K01218 | 1121.333333 | 1121.333333 | 7.275086505 | 0.022419453 |
| K16816 | 13.5 | 13.5 | 27 | 0.006533376 |
| K19551 | 51221.33333 | 51221.33333 | 6.716788474 | 0.026872539 |
| Day 9 |  |  |  |  |
| K01179 | 208012.5 | 208012.5 | 4.848098168 | 0.04269379 |
| K01214 | 35112.5 | 35112.5 | 7.768681896 | 0.013183836 |
| K01732 | 3745.333333 | 3745.333333 | 5.208603746 | 0.045611937 |
| K05349 | 137816.3333 | 137816.3333 | 7.802043312 | 0.019014639 |
| K11424 | 192.6666667 | 192.6666667 | 18.0625 | 0.013158073 |
| K12047 | 33.33333333 | 33.33333333 | 8.928571429 | 0.013618474 |
| K15920 | 120.3333333 | 120.3333333 | 20.98837209 | 0.001008604 |
| K20542 | 5.333333333 | 5.333333333 | 5.714285714 | 0.037928971 |
| K20547 | 50 | 50 | 20 | 0.000385115 |

**Table S5**: Anova results for CPOM-degradation related fungal KEGG Orthologs on day 4, 6 and 9. The variance was measured with aov function from the R package stats on condition (control vs treatment) variable.

| **Day 4** |  |  |  |  |
| --- | --- | --- | --- | --- |
| KEGG_ID | Sum_Sq | Mean_Sq | F_value | Pr_F |
| K00421 | 75525.33 | 75525.33 | 6.36 | 0.03 |
| K01176 | 1294947.00 | 1294947.00 | 6.25 | 0.03 |
| K01179 | 6719778.00 | 6719778.00 | 119.88 | 0.00 |
| K01181 | 310408.33 | 310408.33 | 10.48 | 0.01 |
| K01183 | 31008.33 | 31008.33 | 5.82 | 0.04 |
| K01188 | 47880.33 | 47880.33 | 6.76 | 0.03 |
| K01200 | 41760.50 | 41760.50 | 7.58 | 0.01 |
| K01207 | 1447685.33 | 1447685.33 | 7.06 | 0.02 |
| K01732 | 27840.33 | 27840.33 | 10.74 | 0.01 |
| K05343 | 133141.33 | 133141.33 | 5.00 | 0.05 |
| K05349 | 8151008.33 | 8151008.33 | 8.83 | 0.01 |
| K05350 | 186003.00 | 186003.00 | 7.52 | 0.02 |
| K05909 | 741027.00 | 741027.00 | 6.44 | 0.03 |
| K12309 | 1536.00 | 1536.00 | 17.90 | 0.01 |
| K12373 | 248832.00 | 248832.00 | 5.05 | 0.05 |
| K18337 | 11266.67 | 11266.67 | 22.17 | 0.01 |
| K19551 | 13200.33 | 13200.33 | 45.43 | 0.00 |
| K19668 | 171841.33 | 171841.33 | 17.78 | 0.00 |
| K21574 | 4961.33 | 4961.33 | 5.22 | 0.05 |
| **Day 6** |  |  |  |  |
| KEGG_ID | Sum_Sq | Mean_Sq | F_value | Pr_F |
| K01051 | 4033.33 | 4033.33 | 6.93 | 0.03 |
| K01179 | 3623432.00 | 3623432.00 | 6.67 | 0.02 |
| K01200 | 42050.00 | 42050.00 | 8.32 | 0.01 |
| K01214 | 16370642.00 | 16370642.00 | 6.36 | 0.02 |
| K01218 | 18096.33 | 18096.33 | 26.02 | 0.00 |
| K12047 | 432.00 | 432.00 | 16.96 | 0.00 |
| K12309 | 1441.50 | 1441.50 | 16.14 | 0.02 |
| K19355 | 2465.33 | 2465.33 | 5.39 | 0.04 |
| K19551 | 107541.33 | 107541.33 | 7.25 | 0.02 |
| K19668 | 3034096.33 | 3034096.33 | 8.14 | 0.02 |
| K20547 | 6844.50 | 6844.50 | 15.25 | 0.00 |
| **Day 9** |  |  |  |  |
| KEGG_ID | Sum_Sq | Mean_Sq | F_value | Pr_F |
| K01178 | 320.33 | 320.33 | 5.33 | 0.04 |
| K01732 | 3745.33 | 3745.33 | 5.21 | 0.05 |
| K12047 | 901.33 | 901.33 | 6.34 | 0.03 |
| K18198 | 65.33 | 65.33 | 8.91 | 0.01 |
| K20542 | 5.33 | 5.33 | 5.71 | 0.04 |
| K20547 | 288.00 | 288.00 | 9.14 | 0.01 |
| K20939 | 240.67 | 240.67 | 8.11 | 0.05 |

**Table S6**: ANOVA results for CPOM-degradation related bacterial KEGG Ortholgs on days 4, 6 and 9. The variance was measured with aov function from the R package stats on condition (control vs treatment) variable.

| **Bacteria** | KOs | Functional role |
| --- | --- | --- |
| 1.13.11.79 | K04719 | Sphingobium yanoikuyae NagDI pdo (4-hydroxyphenyl acetate 3-monooxygenase); involved in the degradation of aromatic compounds potentially derived from lignin breakdown. |
| 1.2.7.8 | K00180 | (Formaldehyde ferredoxin oxidoreductase); involved in the oxidation of aldehydes which can play a in lignin breakdown. |
| 2.4.1.187 | K05946 | Cellobiosyl-(1->3)-beta-D-glucosidase; involved in the breakdown of cellulose, an important component of plant cell walls. |
| 3.2.2.10 | K06966 | β-Glucosidase; responsible for breaking down glucosides into glucose, a crucial step in cellulose degradation. |
| **Fungi** |  |  |
| 3.2.1.184 | K18429 | 1. 3.2.1.184: This represents glucuronoxylanase which is involved in the hydrolysis of xylans, a main component of hemicellulose found in plant cell walls. It plays a direct in the breakdown of plant material. |
| 3.2.2.10 | K06966 | β-glucosidase, an enzyme involved in the hydrolysis of terminal, non-reducing β-D-glucosyl residues with release of β-D-glucose. It is crucial in the degradation of cellulose into glucose, directly contributing to the degradation of plant matter |

**Table_S7**: CPOM-related KEGG Ortholog annotation for bacteria and fungi in TCSeq clusters.

**table_S8_diffrential_expression_of_KOs.xlsx**

**Table S8**: Differential analysis of bacterial and fungal significant KOs. significance was implied if the log fold change is a minimum of 2 and the p-adjusted value is maximum of 0.05.

**supplementary_table_S9.xlsx**

**Table S9**: Pathways Enrichment of Significantly expressed KOs for bacteria and fungi. Gene set enrichment of KOs was performed with kegg.gsets from gage R package on KOs which are significant with increased organic matter (control vs treatment).

**supplementary_table_S10.xlsx**

**Table S10**: **Summary of raw Amplicons for 16S and ITS**

| **Fungi** | R2 | F | Pr(>F) |
| --- | --- | --- | --- |
| condition | 0.03365 | 2.0199 | 0.018 * |
| day | 0.15663 | 1.0317 | 0.369 |
| condition:day | 0.1339 | 0.8806 | 0.843 |
| **Bacteria** |  |  |  |
| condition | 0.02466 | 1.4664 | 0.027 * |
| day | 0.16804 | 1.1221 | 0.045 * |
| condition:day | 0.14618 | 0.987 | 0.574 |

---

**Table S11:** PERMANOVA analysis on filtered abundances (read counts greater than 100) of OTUs for ITS1 and 16S rRNA. Adonis2 was used for non-parametric multivariate analysis testing the combination of condition (control vs treatment) and time variables, and their interactions using Bray-Curtis distance metrics. Significance was implied if Pr(>F) is lower than and equal to 0.05 (Signif. codes: 0 ‘***’ 0.001 ‘**’ 0.01 ‘*’ 0.05 ‘.’ 0.1 ‘ ’ 1).

| **Fungi** | baseMean | log2FoldCh | lfcSE | stat | pvalue | padj |
| --- | --- | --- | --- | --- | --- | --- |
| Ascomycota | 1922.86 | 3.19 | 1.32 | 2.42 | 0.02 | 0.03 |
| Basidiomycota | 297.28 | 13.54 | 5.53 | 2.45 | 0.01 | 0.03 |

**Table S12**: Differential analysis of OTUs abundance on phyla level. Abundance was summarised on the phylum level. DESeq2 were employed for the analysis. Significance was implied if the p-adjusted is lower than 0.05 and the log fold change is a minimum of 2.

# References:

Gessner, Mark, and Eric Chauvet. “Qualitative and Quantitative Analyses of Aquatic Hyphomycetes in Streams.” Fungal Diversity Research Series 10 (January 1, 2003)

Gulis, V., Su, R., Kuehn, K.A. (2019). Fungal Decomposers in Freshwater Environments. In: Hurst, C. (eds) The Structure and Function of Aquatic Microbial Communities. Advances in Environmental Microbiology, vol 7. Springer, Cham.<https://doi.org/10.1007/978-3-030-16775-2_5>

Franco-Duarte, R., Fernandes, I., Gulis, V., Cássio, F., & Pascoal, C. (2022). ITS rDNA barcodes clarify molecular diversity of aquatic hyphomycetes. Microorganisms, 10(8), 1569.
